# Four-dimensional flow CMR in tetralogy of fallot: current perspectives

**DOI:** 10.1259/bjr.20210298

**Published:** 2022-02-10

**Authors:** Evangeline Gerdine Warmerdam, Rosalie Lucy Neijzen, Michiel Voskuil, Tim Leiner, Heynric B Grotenhuis

**Affiliations:** Department of Cardiology, University Medical Center Utrecht, Heidelberglaan, Utrecht, The Netherlands; Department of Paediatric Cardiology, University Medical Center Utrecht, Heidelberglaan, Utrecht, The Netherlands; Department of Paediatric Cardiology, University Medical Center Utrecht, Heidelberglaan, Utrecht, The Netherlands; Honours Program Faculty of Medicine, University Medical Center Utrecht, Utrecht, The Netherlands; Department of Cardiology, University Medical Center Utrecht, Heidelberglaan, Utrecht, The Netherlands; Department of Radiology, University Medical Center Utrecht, Heidelberglaan, Utrecht, The Netherlands; Department of Paediatric Cardiology, University Medical Center Utrecht, Heidelberglaan, Utrecht, The Netherlands

## Abstract

Tetralogy of Fallot is the most common cyanotic congenital heart defect, accounting for 10% of all CHD. Despite most patients now surviving well into adulthood, morbidity and mortality rates continue to be high. Surgical and percutaneous pulmonary valve replacement are procedures that are performed to prevent long-term complications from occurring. Unfortunately, pulmonary valve replacement based on current CMR criteria does not prevent postoperative ventricular arrhythmia, heart failure, and sudden cardiac death. Thus, a more advanced and comprehensive hemodynamic evaluation is needed to better understand right ventricular (dys)function in tetralogy of Fallot patients and to optimize the timing of valve replacement. Recently, four-dimensional flow CMR has emerged as a promising and non-invasive imaging technique that can provide comprehensive quantitative evaluation of flow in an entire volume within the chest in a single imaging session. With velocity-encoding in all three spatial directions throughout the complete cardiac cycle, it can provide analysis of cardiac, pulmonary artery and aortic flow volumes, flow velocities, flow patterns, as well as more advanced hemodynamic parameters. Four-dimensional flow CMR could therefore provide insights into the complex hemodynamics of tetralogy of Fallot and could potentially provide novel criteria for pulmonary valve replacement in these patients. The aim of this review is to provide an overview of available research on four-dimensional flow CMR research in tetralogy of Fallot patients.

## Introduction

Tetralogy of Fallot (TOF) is the most common cyanotic congenital heart defect (CHD), accounting for 10% of all CHD.^1^ TOF consists of a combination of a ventricular septal defect (VSD), overriding of the aorta, right ventricular outflow tract (RVOT) obstruction, and right ventricular (RV) hypertrophy.^2^ Surgical repair, consisting of VSD closure and RVOT reconstruction, is usually performed during infancy. Major advances in surgical techniques and perioperative care have resulted in survival rates of over 90% up to 20 years after surgical repair for patients with TOF.^3^ Despite most patients now surviving well into adulthood, morbidity and mortality rates continue to be high during the third and fourth postoperative decades.^3–5^ This decreased survival rate is related to postoperative sequelae such as residual RVOT obstruction, pulmonary regurgitation (PR), and RV dilation or hypertrophy, which ultimately can lead to a negative cascade of RV dysfunction, congestive heart failure, arrhythmias, and sudden cardiac death.^6^

Surgical and percutaneous pulmonary valve replacement (PVR) are procedures that are performed to prevent these long-term complications from occurring. Unfortunately, the indication guidelines for PVR in TOF patients are not clear-cut. With the current cardiac magnetic resonance (CMR) criteria for PVR, such as increased indexed ventricular volumes and reduced ejection fraction, only measures of a late expression of RV dysfunction are taken into account. Although PVR may allow for reduced RV volumes and can improve symptoms, recent research shows that PVR based on current CMR criteria unfortunately does not prevent postoperative ventricular arrhythmia, heart failure, and sudden cardiac death.^7^ Thus, a more advanced and comprehensive hemodynamic evaluation is needed to better understand RV (dys)function in TOF patients and to optimize the timing of PVR in TOF.

Recently, four-dimensional flow CMR (4D flow CMR) has emerged as a promising and non-invasive imaging technique that can provide comprehensive quantitative evaluation of flow in an entire volume within the chest in a single short CMR imaging session. With velocity-encoding in all three spatial directions throughout the complete cardiac cycle, it can provide analysis of cardiac and vascular flow volumes, flow velocities, and flow patterns, as well as more advanced hemodynamic parameters such as kinetic energy, energy loss, helicity, vorticity, and wall shear stress.^8^ With these new advanced flow parameters available, 4D flow CMR could provide insights into the complex hemodynamics of TOF and could potentially provide novel criteria for PVR in TOF patients. The aim of this review is to provide an overview of available research on 4D flow CMR research in TOF patients.

## Imaging in TETRALOGY OF FALLOT

Currently, echocardiography and CMR are the imaging modalities of choice for follow-up of TOF patients. Both modalities can provide a wide range of anatomical and functional parameters, but both also have a number of limitations. Echocardiography can provide information on anatomy and physiology, while qualitative flow assessment can be performed by colour Doppler. Image quality is strongly dependent on the acoustic window and operator skills.^9^ Especially the RV and PAs can be difficult to visualize in older children and adults, given the limited acoustic window due to the retrosternal positioning.

CMR can provide a completely non-invasive three-dimensional evaluation of cardiovascular anatomy, volumes and function. Flow can be analysed using two-dimensional phase-contrast (2D PC) CMR, which can provide flow volumes and velocity measurements perpendicular to a single plane placed in the vessel of interest. To obtain flow measurements each plane of interest has to be individually planned and separate breath-holding scans need to be performed. Furthermore, 2D PC CMR suffers from errors in flow quantification due to motion of the heart relative to the imaging plane and may give an incomplete assessment of blood flow due to technical limitations, especially in complex CHD.

## 4d flow cmr aqcuisition and processing

The term 4D flow CMR is used for time-resolved phase-contrast CMR with flow encoding in all three spatial directions. This scan technique does not require the administration of gadolinium contrast. However, use of gadolinium contrast can enhance the signal-to-noise ratio and improve the velocity-to-noise ratio and enhance the contrast between the blood pool and surrounding tissue.^10^ With 4D flow CMR, all data are acquired in a single free-breathing acquisition. Respiratory gating can be performed using a (diaphragm) navigator or bellows, but several studies demonstrated good agreement between non-gated 4D flow CMR, breath-hold 2D PC CMR and respiratory-gated 4D flow CMR.^11,12^ Both prospective and retrospective ECG gating can be used for cardiac gating in 4D flow CMR. The velocity encoding speed (VENC) is an important scan parameter to be appropriately chosen for correct acquisition of 4D flow CMR, depending on the underlying cardiac condition of interest. Increasing the VENC value leads to a lower signal-to-noise ratio, so it is recommended to choose a VENC that is just above the expected maximum velocity in the area of interest.^8^ In case phase wrapping occurs, antialiasing correction can be used in order to correct or minimize these artefacts.

Dedicated software is required for processing of 4D flow scan data to facilitate visualization and analysis of data. Cardiac and large vessel flow can be visualized using flow vectors, colour-coded streamlines or pathlines, amongst others. Advanced flow parameters can be calculated within a volume of interest and requires segmentation of cardiac and large vessel structures within that volume. Advanced flow parameters such as kinetic energy and wall shear stress are currently only used in a research setting and the clinical significance of these parameters needs to be further investigated.

## Advantages and limitations of 4d flow cmr

**Figure 1.**
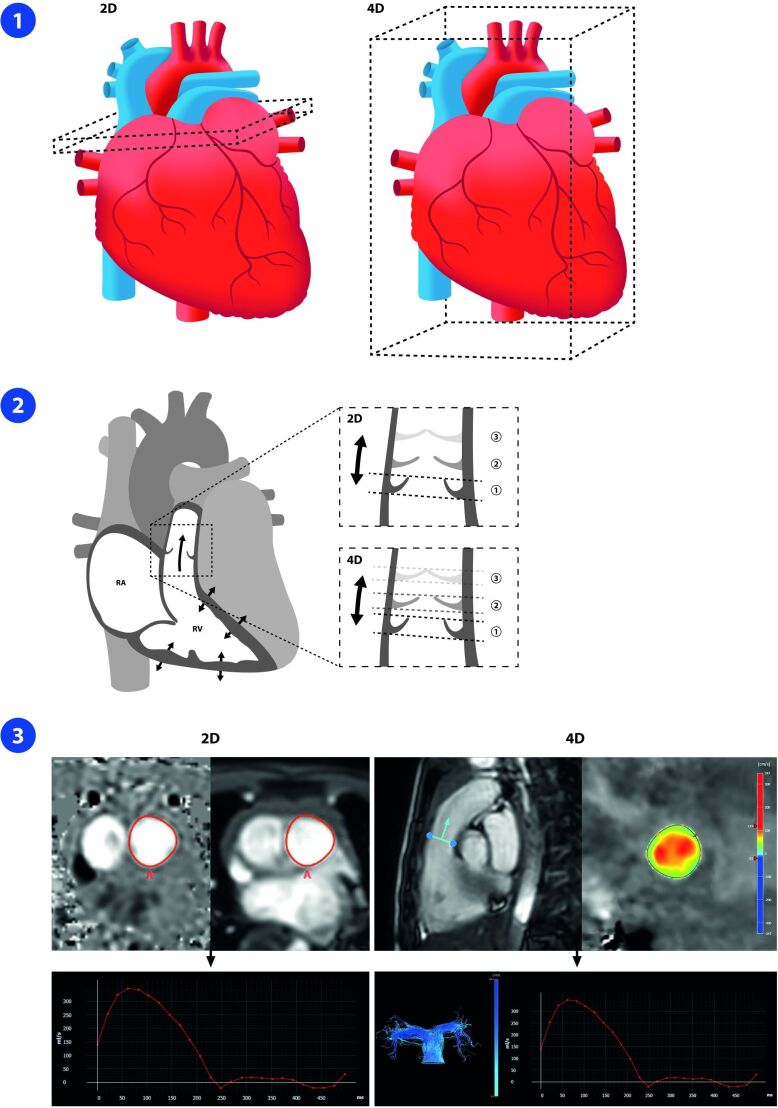
Valve analyses by 2D-PC CMR versus 4D flow CMR. (**A**)For 2D PC CMR, imaging planes for flow measurements have to be individually prescribed, and separate breath-holding scans need to be performed for each acquisition. For 4D flow CMR, all data are acquired in a single acquisition, so any plane of interest can be analyzed retrospectively to study the hemodynamic status of the individual patient. (**B**)In 2D PC CMR, information on valvular blood flow has to be obtained from one static plane, so flow assessment is hampered by the through-plane motion of the valve during the cardiac cycle. 4D flow CMR provides retrospective valve tracking, making it more accurate than 2D PC CMR due to its ability to correct for valvular through-plane motion and flow angulation. (**C**)After identification of the valve area, both 2D PC CMR and 4D flow CMR provide information on basic flow parameters. However, 4D flow CMR can also visualize both the forward and, if present, backward flow over the valve. 2D, two-dimensional; 4D, four-dimensional; CMR, cardiac magnetic resonance; PC, Phase contrast; RA, right atrium, RV, right ventricle. Image from Warmerdam et al.^13^

In general, 4D flow CMR has several advantages over 2D PC CMR. First, any location or plane of interest can be analyzed retrospectively. This is especially relevant in CHD with often complex 3D anatomy. Thus, there is no need for meticulous planning of the acquisition plane and multiple breath-holds as is the case in 2D PC CMR Second, valvular flow can be measured using retrospective valve tracking, which can correct for through-plane motion of the valve and flow angulation (Figure 1).^14^ Flow is measured in all three spatial directions with 4D flow CMR, allowing for qualitative blood flow analysis and providing a wide range of novel advanced flow parameters such as kinetic energy, energy loss, wall shear stress, helicity and vorticity, extending the scope of hemodynamic assessment.

There are several limitations that need to be taken into account when considering implementation of 4D flow CMR in clinical practice. A major drawback is the relatively long acquisition time (up to 10–15 min for a single scan), which can be a burden for the individual patient. Another limitation of 4D flow CMR is the limited spatial and temporal resolution. Currently, 4D flow CMR has a typical spatial resolution of 1.5 × 1.5×1.5–3 × 3×3 mm^3^, and a typical temporal resolution of 30–40 ms.^8^ The accuracy of the measurements of advanced flow parameters is greatly dependent upon the spatial and temporal resolution, and is especially relevant in the younger population. Finally, data processing, visualisation and analysis are more time-consuming and require more skills and dedicated software.

## 4D flow CMR versus 2D PC CMR

The first comparison of 4D flow CMR and 2D PC CMR in patients with TOF was performed by van der Hulst et al (Table 1).^15^ In this study, 4D flow CMR was compared to 2D PC CMR for the evaluation of flow across the pulmonary valve (PV) and tricuspid valve (TV). The investigators found 4D flow CMR to be more accurate in the quantification of forward flow across the TV and regurgitant flow across the PV when compared to 2D PC CMR. Moreover, 4D flow CMR showed better agreement with RV stroke volumes measured in multi slice short axis cine images compared to 2D PC CMR. These results were confirmed in a more recent study by Isorni et al.^16^ In this study, pulmonary and aortic flow measurements by 4D flow CMR and 2D PC CMR were compared for pediatric and adult patients with TOF. Isorni et al found 4D flow CMR to be more reliable and consistent due to the better correlation between pulmonary and aortic flow measurements when compared to 2D PC MRI. Another recent study, by Jakobs et al, also found 4D flow CMR had a better correlation between net flow across the PV and AV when compared to 2D PC CMR.^17^ Overall, 4D flow CMR has consistently shown to be more accurate compared to 2D PC CMR for measuring PV flow and is therefore ideally suited for the evaluation of degree of PR in TOF patients. However, the impact of 4D flow CMR measurements on clinical decision-making however, has yet to be investigated.

**Table 1.** An overview of all available articles, including patient characteristics and results, on four-dimensional flow CMR in tetralogy of Fallot.

First author Year	Patients	Controls	Area of interest	Parameters	Conclusion
Hu 2020	*n* = 25 Age 8.44 ± 4.52 years	*n* = 10 Age 8.2 ± 1.22 years	RV, PAs	Flow, WSS, EL	Increased peak WSS and viscous energy are associated with pulmonary hemodynamic changes.
Jakobs 2020	*n* = 34 Age 15.6 ± 3.6 years	-	PV, PAs	Flow, volumetry	4D flow CMR is more accurate than 2D PC CMR for flow measurements.
Isorni 2019	*n* = 50 Age 18.2 (2–54) years	-	PAs, aorta	Flow	4D flow CMR is more accurate than 2D PC CMR for flow measurements.
Rizk, 2019	*n* = 37 Age 27 (18-35) years	*n* = 11 Age 26 (24-27) years	PAs	WSS	WSS is increased in patients with PS.
Robinson 2019	*n* = 21 Age 13.8 ± 8.2 years	*n* = 24 Age 15.8 ± 3.0 years	RV, PAs	KE	KE is abnormal in TOF patients and has a direct relationship with traditional measures of disease severity.
Schäfer 2019	*n* = 41 Age 14 (10-21) years	*n* = 15 Age 10 (10-18) years	Aorta	EL	Abnormal aortic flow patterns are associated with increased EL. EL is associated with LV volumes and function.
Frederiksson 2018	*n* = 17	*n* = 10 Age 31 ± 11 yearts	RV	Flow, TKE	RV TKE is increased in TOF patients. RV TKE has a stronger association with RV remodeling than 2D PC CMR measurements.
Schäfer 2018	*n* = 18 Age 10.3 ± 3.2 years	*n* = 18 Age 11.6 ± 3.4 years	Aorta	Flow, WSS	TOF patients have increased aortic WSS, increased aortic stiffness and abnormal aortic flow during systole.
Sjöberg 2018	*n* = 15; Age 29 ± 12 years	*n* = 14 Age 30 ± 7 years	LV and RV	KE	TOF patients with PR>20% and preserved LV function have disturbed KE in the LV and RV.
Hirtler 2016	*n* = 24; Age 11.7 ± 5.8 years	*n* = 12 Age 23.3 ± 1.6 years	RV, PAs	Flow, vorticity	TOF patients have increased RV vorticity. Higher PR fraction is associated with increased RV vorticity.
Jeong 2015	*n* = 10 Age 20.6 ± 12.2 years	*n* = 9 Age 38.9 ± 15.1 years	LV, RV, PAs, aorta	KE	TOF patients have increased RV KE.
Francois 2012	*n* = 11 Age 20.1 ± 12.4 years	*n* = 10 Age 34.2 ± 13.4 years	SVC, IVC, RV, PAs	Flow, WSS	TOF patients have increased vortical flow in the RA and RV and have increased helical and vortical flow in the PAs.
Geiger 2011	*n* = 10 Age 12.1 ± 8.1 years	*n* = 4 Age 26 ± 0.8 years	PAs, aorta	Flow, vorticity	TOF patients can have helical flow and severe vortices in the PAs.
Van der Hulst 2010	*n* = 25 Age 13.1 ± 2.7 years	*n* = 19 Age 14.1 ± 2.4 years	TV, RV, PV	Flow	4D flow CMR is more accurate than 2D PC CMR for valvular flow measurements and evaluation of RV diastolic function.

2D PC CMR, two-dimensional phase-contrast cardiac magnetic resonance; 4D flow CMR, four-dimensional flow cardiac magnetic resonance; EL, energy loss; IVC, inferior caval vein; KE, kinetic energy; LV, left ventricle; PA, pulmonary artery; PV, pulmonary valve; RV, right ventricle; SVC, superior caval vein; TV, tricuspid valve; WSS, wall shear stress.

## Right ventricle

Functional assessment of the RV can be challenging due to its complex shape and often dyssynchronous contraction pattern. Since RV function is often impaired in TOF patients, accurate evaluation of RV function is crucial. Multislice short axis images obtained with routine CMR scans can provide routine parameters to assess RV function, such as ejection fraction, end-diastolic volume, and end-systolic volume. Indexation for body surface should be performed in the paediatric population, to correct for body composition and size. The aim of PVR is improvement of RV dimensions and function, which, according to current guidelines, can be achieved when preoperative RV end-diastolic volumes are <160 mL/m^2^ or RV end-systolic volumes are <82 mL/m^2^.^18^

**Figure 2.**
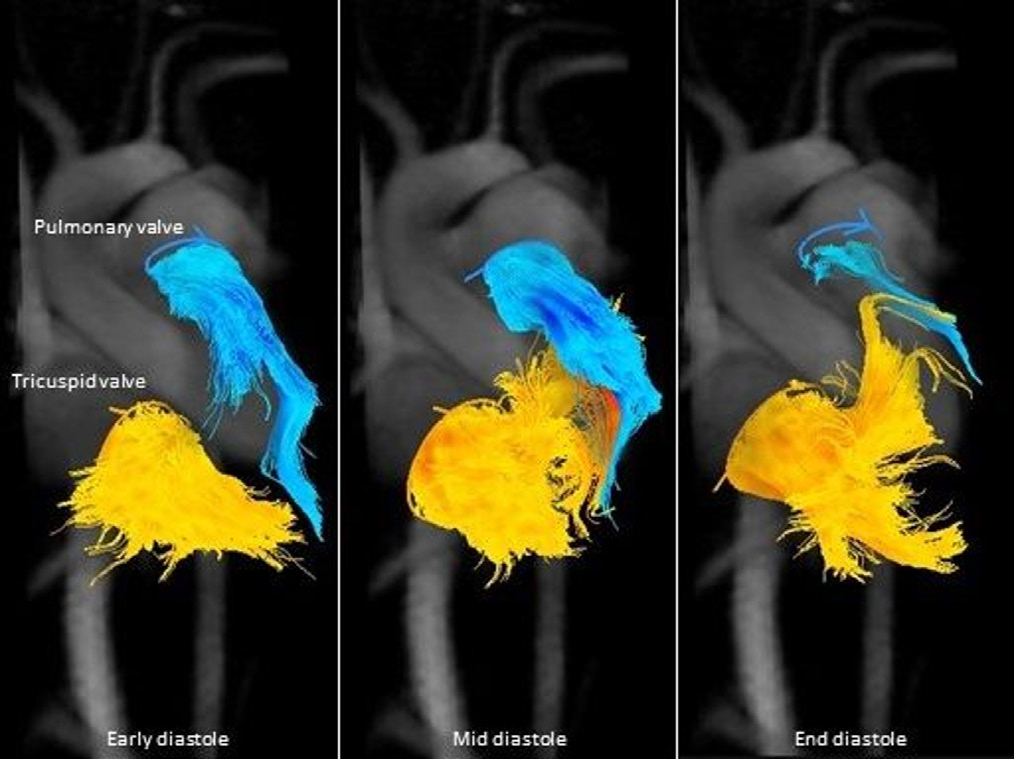
Visualization of flow through the tricuspid valve (orange) and the pulmonary valve (blue). It is clear that the severe pulmonary regurgitation impairs the normal inflow of blood through the tricuspid valve in the right ventricle.

Several studies have investigated flow patterns and advanced flow parameters in the RV using 4D flow CMR. Francois et al found increased vortical flow in TOF patients compared to controls in the right atrium and RV during diastole.^19^ Similar results were found by Hirtler et al, as they found that the degree of PR (Figure 2) was associated with a higher degree of vorticity in the right atrium and the RV.^20^ These right sided vortices may represent energy loss and a less efficient circulation, which can potentially be harmful for the RV in TOF with impaired contractile capacity and could potentially serve to refer patients for early intervention.

One of the advanced parameters that can be measured with 4D flow CMR is kinetic energy, which is the mean energy content per mass unit blood flow. Kinetic energy measurements represent the amount of energy present in the blood flow due to motion and is suggested to be a good marker of ventricular efficiency. Jeong et al used 4D flow CMR to investigate flow energetics in the RV in TOF patients and healthy controls. They found kinetic energy in the RV to be increased in the TOF patients compared to healthy controls.^21^ Similar results were found in two other studies by Sjöberg et al and Frederiksson et al.^22,23^ Sjöberg et al, who evaluated RV kinetic energy over the entire cardiac cycle in patients with TOF with moderate-to-severe PR (>20%) compared to healthy controls. They found RV diastolic peak kinetic energy to be increased in TOF patients compared to healthy controls, especially in patients with non-restrictive RV physiology.^22^ Frederiksson et al found increased turbulent kinetic energy in the RV of patients with TOF and PR compared to healthy controls. High values of turbulent kinetic energy were located predominantly in the RVOT. Furthermore, they found that turbulent kinetic energy had a stronger relationship to indices of RV remodelling, as expressed by PR volumes and fractions, compared to conventional 2D MRI parameters such as RV dimensions.^23^ These findings of increased kinetic energy in the RV suggest that circulation in the RV is less efficient in TOF patients compared to healthy controls. Moreover, kinetic energy could be potentially serve as an important indicator of RV performance, which is often impaired in patients with TOF due to chronic volume overload.

## Pulmonary arteries

**Figure 3.**
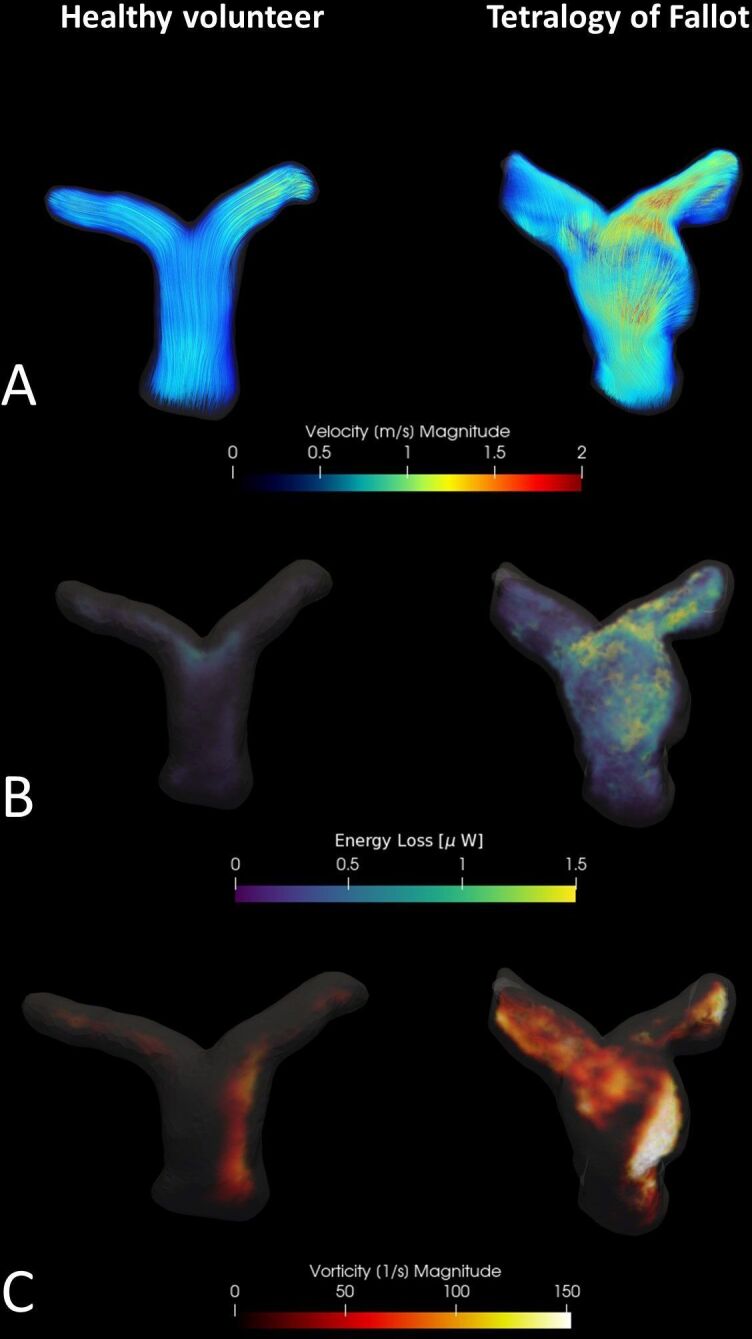
4D flow CMR visualisation of the pulmonary arteries from a healthy volunteer and a TOF patient. Panel A shows flow patterns and flow velocities using colour-coded streamlines. The TOF patient shows dilatation of the main pulmonary artery and clear areas of increased flow velocity and abnormal flow patterns. Panel B shows a visual representation of the energy loss, a possible marker of efficiency of the circulation, within the pulmonary arteries. In healthy pulmonary arteries with normal flow patterns, only a minimal amount of energy loss is seen just before the bifurcation. For the TOF patient, increased levels of energy loss are seen, especially in the areas with non-laminar flow and increased velocity. Panel C shows a visual representation of vorticity, a parameter describing the local flow rotation, in the pulmonary arteries. The TOF patients clearly has higher vorticity values compared to the healthy volunteer. Overall, the TOF patient shows abnormal flow patterns and clearly has a less efficient pulmonary circulation compared to the healthy volunteer.

Patients with TOF can suffer from varying degrees of PR, PV stenosis and PA branch stenosis. Therefore, flow in the PAs can exhibit complex patterns (Figure 3). As a consequence, PA flow analysis based on a single 2D PC CMR plane is often incomplete and prone to errors. Geiger et al and Francois et al have used 4D flow CMR to investigate blood flow patterns in the PAs. They found that TOF patients can exhibit helical flow patterns in the PAs and frequently demonstrate extensive vortex formation.^19,24^ Hu et al performed an analysis of helical and vortical flow patterns in the PAs of TOF patients.^25^ They found vortices to be present predominantly in the main PA, which is most likely related to increased main PA diameters as a result of prior surgical repair. Helical flow patterns were mainly found in the right PA. The mechanism underlying helical flow is not very well understood, but it has been postulated to be a more efficient way of driving blood through a stenotic vessel.^26^ Furthermore, Hu et al found systolic energy loss in the right PA to be associated with increased RV dimensions, which is suggestive of impaired ventricular-arterial coupling. In order to investigate whether 4D flow MRI measurements of hemodynamic inefficiency are associated with disease severity in TOF patients, Robinson et al compared 4D flow MRI measurements to conventional MRI parameters used for the monitoring of disease progression such as RV volumes and ejection fraction.^27^ The researchers found that kinetic energy in the PAs was increased in TOF patients compared to healthy controls and that these kinetic energy measurements were correlated with traditional measurements of disease severity, such as ventricular volumes and the degree of PR. Especially interesting were the strong and positive correlations between increased kinetic energy in the PAs and larger RV dimensions. With 4D flow CMR, parameters of hemodynamic (in)efficiency in the PAs can be measured, and these parameters could therefore serve as early markers of disease progression in TOF patients and, potentially, earlier intervention.

## Left ventricle and aorta

Although follow-up of TOF is primarily focused on the right heart, the status of the left heart also needs to be considered during follow-up of these patients. A substantial number of TOF patients suffer from aortic root dilatation and aortic stiffness, which can result in volume or pressure overload of the LV, respectively.^28^ Three studies have investigated the LV and aorta in patients with TOF using 4D flow CMR. Sjoberg et al investigated LV kinetic energy over the entire cardiac cycle in TOF patients with moderate to severe PR and healthy controls.^22^ They found a significant decrease in LV systolic kinetic energy in TOF patients compared to healthy controls. Possible explanations could be decreased preload of the LV due to RV impairment, septal dyssynchrony (with the septum moving towards the RV during systole), and adverse RV-LV interaction in the setting of RV dysfunction. Schäfer et al used 4D flow CMR to assess aortic wall characteristics and aortic flow hemodynamics in pre-adolescent and adolescent TOF patients who underwent early repair (age <1 year) and compared these findings to age-matched controls.^29^ They found elevated wall shear stress values throughout the entire aorta and increased aortic stiffness in the ascending and proximal descending aorta. In a different study, Schäfer et al found abnormal helical flow patterns in the aorta in TOF patients, whereas none of the control patients showed abnormal flow patterns. In TOF patients, these abnormal flow patterns were found to be associated with increased energy loss and this energy loss was subsequently found to be associated with reduced LV function and volumes.^30^ These findings suggest LVOT abnormalities due to initial TOF repair could result in abnormal flow patterns in the aorta which in turn could lead to impaired LV function.

## Future perspectives

4D flow CMR enables comprehensive evaluation of blood flow within the cardiopulmonary circulation of patients with repaired TOF. In studies comparing 4D flow CMR to 2D PC CMR, 4D flow CMR has been found to be more accurate than 2D PC CMR for measurements of valvular flow, most likely due to ability to correct for through-plane motion of the valve and flow angulation. Since precise evaluation of PR is important, evaluation of valvular flow in TOF patients is better performed using 4D flow CMR. Abnormal flow patterns can be visualized with 4D flow CMR and this can be insightful for both physicians and patients. Furthermore, advanced flow parameters such as vorticity and helicity provide the opportunity to precisely quantify these abnormal blood flow patterns. Further research could help to provide insights into the complex interplay between vessel anatomy and blood flow patterns. Advanced flow parameters that can better assess the hemodynamic consequences of altered postoperative anatomy as well as improved prediction of which patient will develop valvular or arterial stenosis would be of great use in determining follow-up frequency and identifying patients at risk of reintervention. Advanced flow parameters that are possible markers of efficiency of the circulation, such as kinetic energy and energy loss, have been found to be abnormal in TOF patients compared to healthy controls. The above-mentioned research suggests that these parameters could potentially serve as markers for disease progression or even become predictors of cardiovascular outcome.

Timing of PVR in TOF patients has been subject of academic debate for decades. Unfortunately, recent research shows that, using current CMR criteria, PVR does not prevent postoperative ventricular arrhythmia, heart failure, and sudden cardiac death in a significant portion of patients.^7^ Using 4D flow CMR, a more accurate assessment of PV regurgitation flow can be obtained, which could lead to better timing of PVR. Furthermore, 4D flow CMR provides a wealth of advanced flow parameters which could be used to assess ventricular and vascular function in TOF patients. Research using 4D flow CMR in large cohorts of TOF patients with a long follow-up period could potentially validate these novel indicators in decision making for PVR (Table 2).

**Table 2.** A summary of merits of four-dimensional flow cardiac magnetic resonance in patients with tetralogy of Fallot.

Advantages of 4D flow CMR in TOF
- More accurate assessment of pulmonary valvular flow
- Visualization of blood flow in pulmonary artery and right ventricle
- Advanced hemodynamic parameters for ventricular and vascular function assessment
- Potentially novel hemodynamic parameters to further refine indication and timing of pulmonary valve replacement

4D flow CMR, four-dimensional flow cardiac magnetic resonance; TOF, tetralogy of Fallot.

Thus, 4D flow CMR provides a unique opportunity to investigate abnormal blood flow in TOF due to (repaired) defects and study flow phenomena and arterial-ventricular interaction. Further research could potentially identify parameters suitable for the prediction of outcomes in patients with repaired TOF and for refining timing of PVR.

## Conclusion

A substantial body of research shows 4D flow CMR is more accurate in measuring pulmonary flow and especially valvular regurgitation fraction compared to 2D PC CMR. Furthermore, 4D flow CMR provides the opportunity to analyze a wide range of advanced hemodynamic parameters not accessible with any other method. Compared to healthy controls, patients with TOF have increased kinetic energy and vortex flow in the RV and PAs and increased wall shear stress and abnormal flow patterns in the aorta, all of which are indicative of a less efficient circulation. Further research into advanced flow parameters in patients with TOF could potentially validate parameters suitable for prediction of outcomes in patients with repaired TOF and for refining timing of PVR.
